# Smartphone addiction and cross-cultural adjustment among overseas Chinese students: The role of emotion regulation beliefs and strategies

**DOI:** 10.3389/fpsyg.2022.1009347

**Published:** 2022-10-10

**Authors:** Huang Wanqing, Liang Fenqing, Alexander Solodukho

**Affiliations:** ^1^Department of Social and Organizational Psychology, Faculty of Philosophy and Social Science, Belarusian State University, Minsk, Belarus; ^2^Shenzhen Tiantian Brothers Technology Co., Shenzhen, China

**Keywords:** cross-cultural adjustment, smartphone addiction, emotional regulation strategies, emotion regulation beliefs, childhood emotional neglect

## Abstract

**Background:**

Few studies have focused on the cross-cultural adjustment of Chinese students studying in Belarus with the size of this group increasing in recent years. The current study aimed to map the process of cross-cultural adjustment onto various factors including childhood emotional neglect, emotion regulation beliefs, emotional regulation strategies, and smartphone addiction in the international students. Emotional regulation strategy and emotion regulation beliefs could perform as key parts in adapting into overseas life from social learning perspective. Furthermore, smartphone addiction could precipitate a failed adjustment process.

**Materials and methods:**

A total of 356 Chinese students in Belarus completed a self-administered questionnaire including the Chinese versions of the 12-item general health questionnaire (GHQ-12), sociocultural adaptation scale, mobile phone addiction tendency scale for college students, emotion regulation questionnaire, emotion and regulation beliefs scale, and childhood trauma questionnaire-short form. Correlation analysis, regression analysis and *T*-tests were used to explore the relationship between the variables. Structural equation modeling was carried out to test the hypotheses for association.

**Results:**

Emotion regulation beliefs of international students mediated the effect of childhood emotional neglect on cross-cultural adjustment through expression suppression and smartphone addiction. While, in another chain mediation model, childhood emotional neglect affected cross-cultural adjustment only through emotion regulation beliefs and smartphone addiction. Cognitive appraisal independently influenced adjustment through smartphone addiction.

**Limitations:**

Limitations include its cross-sectional design and self-reported survey methodology. In the future, we can combine experimental manipulations to explore the mechanisms by which various emotion beliefs act on smartphone addiction and cross-cultural adjustment in different situations.

**Conclusion:**

This study displays the correlation between emotion regulation beliefs to smartphone addiction and cross-cultural adjustment, as well as the harmful effects of childhood emotional neglect; these components should be further addressed in future studies.

## Introduction

The number of Chinese students studying abroad has been increasing in recent decades. The data show that the total number of Chinese international students in 2019 was 703,500 (Moe.gov.cn, 2022). In the academic year 2020–2021 more than 7,000 Chinese students studying in Belarus (Ministry of Education of the Republic of Belarus, 2022) and more than 30,000 Chinese students studying in Russia (Ministry of Science and Higher Education of the Russian Federation, 2022). China has many cultural concepts that are very different from those in the West (Jain, 2006). For example, Chinese tend to hold more collectivist attitudes than Russians (Tu et al., 2011), which can lead a more indirect way in communication (Gulbro and Herbig, 1999) and may affect the cross-cultural adjustment of Chinese students in Russian-speaking regions.

University students are in the stage of emerging adulthood, a period of high prevalence of mental health disorders (Arnett et al., 2014). Studying abroad gives international students the opportunity to receive a high level of education, but it also raises a variety of mental health issues. In February 2022, the situation in Ukraine deteriorated. According to Reuters (2022), there were casualties among foreign students in the eastern Ukrainian city of Kharkiv. The Russian–Ukrainian conflict has affected the mental and emotional state of university students in the region, with most reporting warfear about the war, stress, and angere (Kurapov et al., 2022). In addition, COVID-19 is spreading rapidly in Eastern Europe and this pandemic can have an impact on international students’ anxiety, stress, and concerns about academic delays (Konstantinov et al., 2022). The unstable regional situation and the outbreak of COVID-19 may have a double mental impact on international students at the emerging adulthood. In the current crisis, it is of practical and theoretical importance to research the mechanisms influencing the cross-cultural adjustment of international students in order to promote the mental health of this group.

### Cross-cultural adjustment

Cross-cultural adjustment includes psychological adaptation and sociocultural adaptation of individuals in a new social environment (Searle and Ward, 1990; Ward and Kennedy, 1994). Psychological adaptation refers to psychological and emotional wellbeing in cross-cultural encounters, and sociocultural adaptation refers to the ability to adapt to the local sociocultural environment (Ward and Kennedy, 1992). Previous studies have explored cross-cultural adjustment from the perspectives of personality, cultural intelligence and social support (Wang et al., 2015; Bender et al., 2019; Hu et al., 2020), but there is little research to uncover the capability of emotion regulation in the cross-cultural adjustment of international students.

Social cognitive theory emphasizes the importance of cognitive factors played in between the environment stimulus and behavior (Bandura, 1997). This theory suggests that there is a dynamic, triangle among cognition, environment and behavior. Based on social cognitive theory, individuals may develop maladaptive emotion beliefs as a result of experience of emotional neglect in childhood. These beliefs may affect their emotion regulation strategies in adulthood and eventually lead to cross-cultural adjustment problems.

Only a few studies investigated cross-cultural adjustment of international students in Russian-speaking regions. Shi conducted a survey of Chinese international students in Kyrgyzstan, and the results showed that the majority of Chinese international students suffered from varying degrees of depression (Shi, 2019). A survey of 351 international students in Russia showed that Chinese students had less adjustment skills and higher levels of depression compared to international students from other countries (Ju, 2011). However, there are barely studies focusing on the cross-cultural adjustment of Chinese students in Belarus. Therefore, the current study aims to investigate the factors influencing cross-cultural adjustment of Chinese students in Belarus from the social cognitive theory perspective. This could further contribute to the potential intervention to help international Chinese students to better adapt into the local culture.

### Smartphone addiction

In the digital era, while smartphones bring convenience to international students, over-reliance on smartphones can also negatively affect their physical and psychological health. A study conducted in Korea in 2015 reported that 40% of the Chinese international students were at-risk smartphone users (Kim et al., 2015). There are no studies have focused on smartphone addiction among international students in Russian-speaking regions.

Symptoms of smartphone addiction include “inability to control cravings,” “anxiety and feeling lost,” “withdrawal and escape,” and “productivity loss” (Leung, 2008). Previous studies showed that smartphone addiction is associated with problems such as depression, anxiety, and realistic social avoidance (Elhai et al., 2017; Rozgonjuk et al., 2018; Wu et al., 2019), which may trigger a range of maladaptation problems in individuals. Despite the many negative effects that smartphone addiction may have on individuals’ social adjustment, few studies have focused on the mechanisms underlying the international students’ cross-cultural adjustment and the role played by smartphone addiction in this process.

### Emotional regulation strategies

Expressive suppression and cognitive reappraisal are two commonly used emotional regulation strategies that are widely associated with individuals’ psychological health. Expression suppression is a response-focused strategy that impedes individual emotional expression through response adjustment; cognitive reappraisal is an antecedent-focused strategy that alters emotional responses by changing the interpretation of the situation (Gross, 1998). It has been suggested that expression suppression and cognitive reappraisal act independently (Wang et al., 2007). Evidence from electrophysiological (EEG) and event related potential (ERP) suggests that different neurophysiological mechanisms exist for these two emotional regulation strategies (Sun et al., 2020).

Emotional regulation ability is an important factor influencing cross-cultural adjustment (Matsumoto et al., 2007). However, there are inconsistencies in the previous research findings. A survey of 245 international students in China during the epidemic found that psychological problems such as fear and hypochondriasis were significantly and negatively associated with both emotional regulation strategies (Xv et al., 2022). Interestingly, another survey of Chinese students in Ireland, however, found that expression suppression was not associated with poor psychological functioning in the Chinese student population (Sun and Nolan, 2021). In light of this, it is necessary to further discuss the relationship between expression suppression and cognitive reappraisal and international students’ cross-cultural adjustment.

The way individuals regulate their emotions may have an impact on the consequences of their smartphone use (Fortes et al., 2021). It has been shown that expression suppression positively predicts smartphone use problems (Rozgonjuk and Elhai, 2021), cognitive reappraisal is positively associated with internet addiction (Trumello et al., 2018). Meanwhile, some researchers concluded that both expression suppression and cognitive reappraisal were significantly and negatively associated with smartphone addiction (Zhang and Jiang, 2017). On the other hand, other researchers emphasized that no emotional regulation strategy is beneficial or harmful under all the circumstances (Haines et al., 2016; Ford et al., 2019). Additionally, previous studies barely focused on the effect of emotional regulation strategies on smartphone addiction in international students.

### Emotion regulation beliefs

Emotion regulation beliefs are the beliefs about whether emotions can be regulated, and they shape the individual’s tendency toward emotion regulation strategies (Romero et al., 2014; Schroder et al., 2015), and may influence individuals’ long-term development. Adults who tend to hold emotionally unregulated beliefs suffer from deteriorated mental health (De Castella et al., 2014; Ford et al., 2018).

Emotion regulation beliefs are related to an individual’s tendency to use emotion regulation strategies. Several cross-sectional studies found that individuals’ emotionally unregulated beliefs were not related to expression suppression, but were related to cognitive reappraisal (De Castella et al., 2013; Schroder et al., 2015). However, there is also evidence that individuals who hold emotionally unregulated beliefs are more likely to use expression suppression to regulate negative emotions (Wang, 2016). Ford and Gross noted that emotion regulation beliefs about experience may influence the choice of cognitive reappraisal, and emotion regulation beliefs about expression may influence the choice of expression suppression (Ford and Gross, 2018). Given the inconsistent findings of the previous studies, an in-depth discussion of the relationship between emotion regulation beliefs and emotional regulation strategies is necessary.

There is evidence that individuals who tend to believe that their emotions are unregulated have lower levels of social adaptation, and if individuals believe that they can regulate their emotions, they will have higher levels of social adaptation (Tamir et al., 2007). Therefore, emotion regulation beliefs may affect individuals’ cross-cultural adjustment, and international students need to have high levels of emotion regulation beliefs in order to successfully complete cross-cultural adjustment.

Some studies have suggested that negative beliefs are markers of addictive behaviors (Hamonniere and Varescon, 2018). A person’s negative beliefs may lead to problematic internet use behaviors (Spada and Caselli, 2017). Specifically, emotion beliefs predict symptoms of emotion dysregulation (Veilleux et al., 2021), and difficulties with emotion regulation are positively predictive of smartphone addiction (Ye et al., 2017). However, no studies have focused on the role of emotion regulation beliefs in smartphone addiction and cross-cultural adjustment in international students.

### Childhood emotional neglect

Childhood emotional neglect can have a negative impact on the individual (Cohen et al., 2017; Müller et al., 2019; Salokangas et al., 2019). Among the types of childhood maltreatment, emotional neglect is an implicit form of maltreatment that refers to the failure of caregivers to meet the emotional needs of children for normal development (Gilbert et al., 2009). It is estimated that approximately 18% of children worldwide suffer from emotional neglect each year (Stoltenborgh et al., 2015). A survey of adolescents in Henan, China, found that the prevalence of emotional neglect during childhood was 53.51% (Yu et al., 2020), which is higher than global estimates of the rate of emotional neglect in childhood. Because childhood emotional neglect occurs during a critical period of brain development, these adverse experiences may lead to changes in the structure and function of the human brain (Frodl et al., 2010; Maheu et al., 2010; Womersley et al., 2020), which subsequently affects the long-term health status of individuals.

Individuals’ social adjustment and psychological wellbeing in adulthood can be affected by childhood emotional neglect (Wang et al., 2019). A study by Popescu et al. (2010) showed that adverse childhood experiences can affect social functioning in adulthood through individuals’ coping styles. Although these studies point out that childhood emotional neglect can negatively affect individuals’ adaptive development in adulthood, there is no research linking childhood emotional neglect to cross-cultural adjustment in international students, and it is necessary to explore the mechanisms at play.

Childhood emotional neglect is associated with the patterns in which individuals cope with their emotions. Previous research has shown that children as young as 6 years old have already formed reliable beliefs about specific forms of emotion regulation (Waters and Thompson, 2014). Young’s schema theory proposes that adverse childhood experiences, contribute to individuals forming early maladaptive schemas about self and others (Young et al., 2003), also known as maladapted cognition. An individual’s physical and psychological health can be affected by maladapted cognitions in a lasting way. However, to our knowledge, little research has been conducted to date on the relationship between childhood emotional neglect and emotion beliefs.

### The current study

**FIGURE 1:**
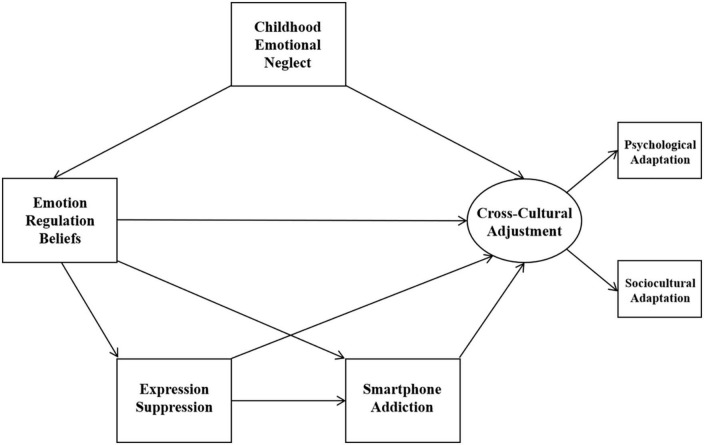
Model 1.

**FIGURE 2:**
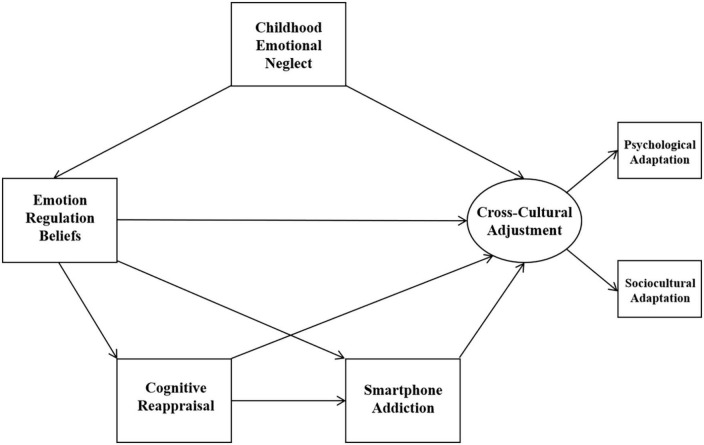
Model 2.

In sum, the previous studies have rarely explored the relationship between childhood emotional neglect, emotion regulation beliefs, emotional regulation strategies, smartphone addiction, and cross-cultural adjustment within a broad framework. The present study focused on two emotional regulation strategies, expression suppression and cognitive reappraisal. Given the possible differences in the mechanisms of action of these two emotion regulation strategies on smartphone addiction and cross-cultural adjustment in international students, and the fact that their relationship with individual psychological responses has not yet reached a consistent conclusion. Therefore, we proposed two models using social cognitive theory as the underlying framework (Liu and Ling, 2009). In these two models, we predicted that emotion regulation beliefs would mediate the relationship between childhood emotional neglect and cross-cultural adjustment (Hypothesis 1). Next, we predicted that emotional regulation strategies would mediate the relationship between emotion regulation beliefs and smartphone addiction (Hypothesis 2). Furthermore, we predicted that emotional regulation strategies would mediate the relationship between emotion regulation beliefs and cross-cultural adjustment (Hypothesis 3). Finally, we predicted that smartphone addiction would mediate the relationship between emotion regulation strategies and cross-cultural adjustment (Hypothesis 4). The conceptual models are shown in Figures 1, 2.

## Materials and methods

### Study design and sample

The survey, based on a cross-sectional design, was conducted in Belarus in March 2022. Participants completed questionnaires online via *Wenjuanxing*.^1^ All participants received a link to the questionnaire on their smartphones, which was posted on a WeChat group of Chinese students in Belarus. The questionnaire can be found in the Supplementary material. Participants first signed an informed consent form and then completed and submitted a questionnaire, and finally all participants received RMB 5 as incentives. The survey is completely anonymous and confidential and takes around 3–5 min to complete. The study was approved by the Ethics Committee of the Belarusian State University and was conducted according to the American Psychological Association guidelines in accordance with the 1964 Helsinki Declaration. The survey was preset up so that it could not be submitted without being totally completed, which helped to avoid the problem of missing values. Prior to data analysis, 15 invalid questionnaires (*n* = 371) were excluded because of (1) repeated submission; or (2) completing the entire questionnaire in 180 s and not understanding each question properly; or (3) choosing completely different answers to questions with similar meaning (For example: the truth is, I have difficulty controlling my emotions/no matter how hard I try, I have difficulty controlling my emotions). Thus, our final sample consisted of 356 Chinese students in Belarus, with a valid response rate of 95.96%.

### Demographic characteristics of participants

Information on demographic data was gathered including age, gender, education level, number of siblings, parents’ marital status, socioeconomic level, duration of stay in Belarus, and academic performance.

### General health questionnaire-12 item

The 12-item general health questionnaire (GHQ-12) was used to measure the participants’ psychological adaptation (Goldberg, 1978). The GHQ-12 consists of 12 Likert-style items using a 0-0-1-1 scoring system, ranging from “0” (not at all) to “1” (much more serious than usual), with a total score ranging from 0 to 12. The optimal cut-off score is 4. A score greater than or equal to 4 means that the participant is positive for mental disorder screening, i.e., poorly psychological adaptation, while a score less than 4 means that the participant is negative for mental disorder screening, i.e., well psychological adaptation. The reliability of the Chinese version of the GHQ-12 has been confirmed (Yang et al., 2003). The Cronbach’s Alpha coefficient for this scale was 0.792.

### Sociocultural adaptation scale

Sociocultural adaptation was measured by 15 items taken from the original 29 items of sociocultural adaptation scale (Ward and Kennedy, 1999). The scale consists of 15 Likert-type items scored on a five-point scale, ranging from “1” (extremely difficult) to “5” (no difficulty), with a total score ranging from 15 to 75, with scores approaching 75 indicating a better sociocultural adaptation of the respondent. This scale is adaptable and easily modified in terms of cultural appropriateness in a variety of research environments (Ward and Rana-Deuba, 1999). Tao adapted some of the SAS statements to match Chinese culture, for example, the items “worship” and “political system” were dropped as potentially misleading (Tao, 2012). The adapted version was used as the measurement instrument in this study. The Cronbach’s Alpha coefficient for the scale was 0.905.

### Mobile phone addiction tendency scale for college students

The mobile phone addiction tendency scale for college students was used to measure the participants’ level of smartphone addiction (Xiong et al., 2012). The scale consists of 16 Likert-type items on a five-point scale, ranging from “1” (very unlikely) to “5” (fully likely), with a total score ranging from 16 to 80. The higher the score, the higher the level of smartphone addiction for that participant. The Cronbach’s Alpha coefficient for this scale was 0.915.

### Emotion regulation questionnaire

The emotion regulation questionnaire was used to measure participants’ tendency to regulate their emotions (Gross and John, 2003). The scale consists of two dimensions (expression inhibition and cognitive reappraisal), with 10 Likert-type items on a seven-point scale, ranging from “1” (strongly disagree) to “7” (strongly agree), with a total score ranging from 10 to 70. The higher the score, the more often the participant used emotional regulation strategies. The reliability and validity of the Chinese version of this scale were confirmed to be at a reasonable level (Zhao et al., 2015). The Cronbach’s alpha coefficients for the two dimensions of the questionnaire were 0.868 and 0.733, respectively, and the total Cronbach’s alpha coefficient was 0.851.

### Emotion and regulation beliefs scale

The emotion and regulation beliefs scale was used to measure participants’ emotion regulation beliefs (Veilleux et al., 2015). The scale consists of 16 Likert-type items on a five-point scale, ranging from “1” (strongly disagree) to “5” (strongly agree), with total scores ranging from 16 to 80. The higher the score, the stronger the emotion regulation beliefs of that participant. Zhang adapted the ERBS to suit Chinese culture, for example, replacing “acknowledge” with “expression” in entry 11, and deleting three entries with a correlation coefficient of less than 0.30 with the total score (Zhang, 2018). The Cronbach’s Alpha coefficient for the scale was 0.861.

### Childhood trauma questionnaire-short form

The emotional neglect dimension of the childhood trauma questionnaire-short form was used to measure the degree of emotional neglect experienced by participants in childhood (Bernstein et al., 1998). The emotional neglect dimension consists of five Likert-type items scored on a five-point scale, ranging from “1” (never) to “5” (always), with a total score ranging from 5 to 25. A higher score means that the participant experienced more childhood emotional neglect. The Chinese version of the CTQ-SF has reliable letter validity in a sample of Chinese university students (Zhang, 2011). The Cronbach’s Alpha coefficient for the emotional neglect dimension was 0.791.

### Statistical analysis

Statistical analysis was performed using SPSS 23.0 and statistical significance was determined with a two-tailed probability value of <0.05. The measured variables were tested for common method bias using a Harman one-way test with a 40% threshold criterion (Zhou and Long, 2004). Descriptive analyses were conducted on demographic variables. Correlations between measured variables were assessed using Pearson’s correlations. Linear regression analysis was used to measure the effects of demographic characteristics on the measured variables. *T*-tests were used to examine the differences between different demographic variable groups on each variable. All measured variables involved in this study were standardized.

Mplus 8. 0 software was used for structural equation modeling (SEM) analysis to mediate the data. All the indicators involved in the SEM model had a good fit to the SEM criteria (χ^2^/*df* < 5, RMSEA < 0.08, CFI > 0.90, SRMR < 0.05) (Wheaton et al., 1977; MacCallum et al., 1996; Byrne, 1998; Hu and Bentler, 1999). For the mediation analysis, we extracted 5000 bootstrap samples and calculated 95% confidence intervals for bootstrap (BCa 95% CI) and two-tailed probability values < 0.05 being considered statistically significant. At the first stage of statistical analysis, the Harman one-way test was adapted to test for common method bias. As for that demographic statistics for research groups were calculated. At the second stage of analysis, correlation analysis were used to identify relationships between the variables (not included demographic variables). At the third stage of statistical analysis, a regression analysis was used to examine the prediction effect of demographic variables on “psychological” variables. At the fourth stage of statistical analysis, we used a *T*-test to identify differences across demographic variable groups on “psychological” variables. Finally, we tested hypothetical models using the SEM method.

## Results

### Description of the participants

Information on demographic characteristics is presented in Table 1.

**TABLE 1:** Demographic characteristics.

Variables	Range	Mean (SD)	
Age	18–35	23.45 (3.374)	

**Variables**		***n***	**%**

Gender	Male	180	50.6
	Women	176	49.4
Education level	Below bachelor’s degree	36	10.1
	Bachelor’s degree	158	44.4
	Master’s degree and above	162	45.5
Are an only child or not	Only child	188	52.8
	Non-only child	168	47.2
Parents’ marital status	Parents living together	312	87.6
	Divorced parents	44	12.4
Economic level	Poverty	34	9.6
	Not wealthy	56	15.7
	General	224	62.9
	Wealthy	42	11.8
Duration in Belarus	Under 6 months	93	26.1
	6–12 months	109	30.6
	1–2 years	48	13.5
	2–3 years	52	14.6
	More than 3 years	54	15.2
Academic performance	Bad	25	7.0
	General	133	37.4
	Good	157	44.1
	Excellent	41	11.5

### Common method bias test

The Harman one-way test was adapted to test for common method bias. The results showed that the variance explained by the first common factor was only 12.35%, which was less than the critical value of 40%, indicating that there were no serious problems of common method bias in this study.

### Correlations

The means, standard deviations and binary correlations between the measured variables are shown in Table 2. Smartphone addiction was significantly and positively correlated with expressive suppression and cognitive reappraisal, and significantly and negatively correlated with psychological adaptation, sociocultural adaptation and emotion regulation beliefs. Psychological adaptation was significantly and positively correlated with sociocultural adaptation, emotion regulation beliefs, and cognitive reappraisal, and was significantly and negatively correlated with childhood emotional neglect and expressive suppression. Childhood emotional neglect was significantly and negatively associated with emotion regulation beliefs, cognitive reappraisal, and sociocultural adaptation. Sociocultural adaptation was significantly and positively associated with emotion regulation beliefs and cognitive reappraisal. Emotion regulation beliefs were significantly and negatively associated with expressive suppression. Cognitive reappraisal was significantly and positively correlated with expressive suppression.

**TABLE 2:** Mean, standard deviation and bivariate correlation between measured variables (*n* = 356).

	*M*	SD	1	2	3	4	5	6
(1) Smartphone addiction	47.823	12.919	−					
(2) Psychological adaptation	6.536	2.792	−0.211**	−				
(3) Childhood emotional neglect	10.508	4.003	0.073	−0.149**	−			
(4) Sociocultural adaptation	59.797	10.275	−0.229**	0.281**	−0.215**	−		
(5) Emotion regulation beliefs	48.365	4.753	−0.350**	0.403**	−0.126*	0.212**	−	
(6) Cognitive reappraisal	30.379	5.804	0.202**	0.138**	−0.190**	0.212**	0.048	−
(7) Expressive suppression	17.544	4.522	0.386**	−0.109*	0.020	–0.043	−0.286**	0.434**

*p < 0.05, **p < 0.01.

### Regression analysis

We examined the effect of demographic variables on cross-cultural adjustment using regression analysis. With all the variables entered in the regression model, the academic performance and duration of study abroad associated with psychological adaptation significantly, the academic performance and economic level associated with sociocultural adaptation significantly. The better the academic performance, the higher the degree of psychological adaptation (*B* = 0.174, *p* = 0.001); the longer the duration of study abroad, the higher the degree of psychological adaptation (*B* = −0.156, *p* = 0.003), while the other demographic variables had no significant predictive effect on psychological adaptation. The better the academic performance, the higher the level of sociocultural adaptation (*B* = 0.183, *p* = 0.001); the higher the economic level, the higher the level of sociocultural adaptation (*B* = 0.169, *p* = 0.001), and the other demographic variables were not significant predictors of sociocultural adaptation.

We also examined the predictive effect of demographic variables on expression suppression using regression analysis. With all the variables entered in the regression model, the education level associated with expression suppression significantly. The higher the education of international students, the less they used expression suppression to regulate their emotions (*B* = −0.139, *p* = 0.008), and the other demographic variables were not significant predictors of the use of expression suppression. In addition, demographic variables were not significant predictors of childhood emotional neglect, emotion regulation beliefs, cognitive reappraisal, and smartphone addiction.

### *T*-test

We examined the differences across demographic variable groups on each variable using *T*-tests. Results found that in terms of childhood emotional neglect: participants in families with divorced parents suffered more childhood emotional neglect compared to participants in families with parents living together (*T* = −5.803, *df* = 354, *p* < 0.001), the difference in the degree of childhood emotional neglect was not significant in other groups with different demographic variables. Regarding the propensity to use expression suppression: male participants used expression suppression more often to regulate emotions compared to female participants (*T* = 2.804, *df* = 354, *p* = 0.005), and there were no significant differences in the propensity to use expression suppression across the other different demographic variable groups. In addition, there were no significant differences in the levels of psychological adaptation, sociocultural adaptation, smartphone addiction, cognitive reappraisal, and emotion regulation beliefs across the different demographic variable groups.

### Results of structural equation model

**FIGURE 3:**
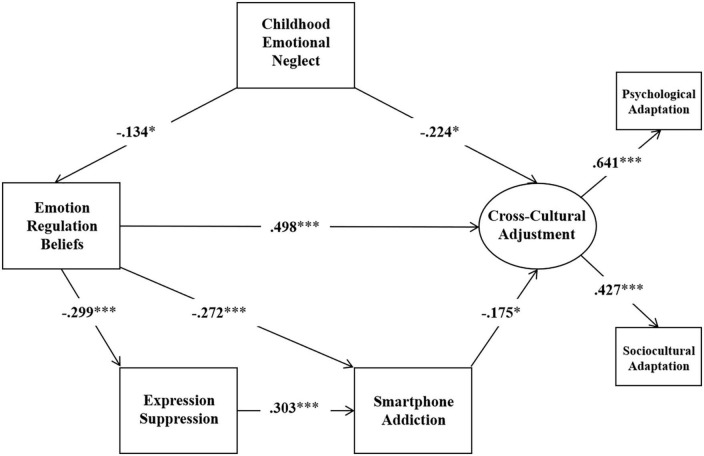
Model 3. *p < 0.05, ^***^p < 0.001.

**FIGURE 4:**
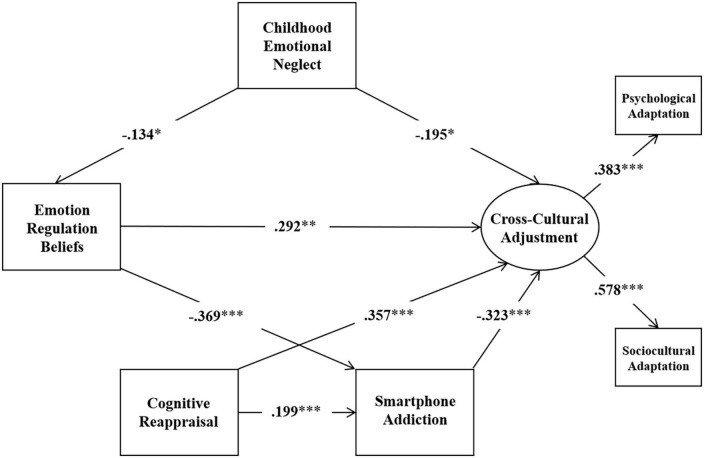
Model 4. *p < 0.05, **p < 0.01, ***p < 0.001.

As planned, the two hypothesized models (as depicted in Figures 1, 2) were examined using SEM method. Results found that Model 1 fit the data well, but Model 2 did not (Model 1: χ^2^/*df* = 2.116, RMSEA = 0.056, CFI = 0.976, SRMR = 0.026; Model 2: χ^2^/*df* = 6.738, RMSEA = 0.127, CFI = 0.840, SRMR = 0.057). However, we found the pathway from expression suppression to cross-cultural adjustment in Model 1 was not significant (*p* > 0.05) and the pathway from emotion regulation beliefs to cognitive reappraisal in Model 2 was not significant (*p* > 0.05). Therefore, we removed the pathway from expression suppression to cross-cultural adjustment in Model 1 and examined the refined model (named Model 3 and depicted in Figure 3). In addition, we removed the pathway from emotion regulation beliefs to cognitive reappraisal in Model 2 and examined the refined model (named Model 4 and depicted in Figure 4). Results show that both the Model 3 and Model 4 fit the data well (Model 3: χ^2^/*df* = 1.961, RMSEA = 0.052, CFI = 0.975, SRMR = 0.028; Model 4: χ^2^/*df* = 3.114, RMSEA = 0.077, CFI = 0.935, SRMR = 0.038).

Further path analyses were conducted to investigate indirect effects. The standardized indirect effect estimate, 95% confidence intervals, relative mediating effects, *p*-values, are shown in Table 3.

**TABLE 3:** Bootstrap-based specific mediation test analysis.

Model	Path	Standardized indirect effects	95% Confidence interval		*P*-value
			Lower limit	Upper limit	
Model 3	Emotion regulation beliefs → Smartphone addiction → Cross-cultural adjustment	0.048	0.006	0.114	0.080
	Childhood emotional neglect → Emotion regulation beliefs → Cross-cultural adjustment	–0.067	–0.136	–0.014	0.029
	Emotion regulation beliefs → Expression suppression → Smartphone addiction → Cross-cultural adjustment	0.016	0.003	0.037	0.055
	Emotion regulation beliefs → Expression suppression → Smartphone addiction	–0.091	–0.143	–0.053	0.000
Model 4	Cognitive reappraisal → Smartphone addiction → Cross-cultural adjustment	–0.064	–0.130	–0.027	0.009
	Emotion regulation beliefs → Smartphone addiction → Cross-cultural adjustment	0.119	0.056	0.205	0.001
	Childhood emotional neglect → Emotion regulation beliefs → Cross-cultural adjustment	–0.039	–0.112	–0.005	0.107
	Childhood emotional neglect → Emotion regulation beliefs → Smartphone addiction → Cross-cultural adjustment	–0.016	–0.041	–0.004	0.069

### Mediation effects from childhood emotion neglect to cross-cultural adjustment through emotional regulation beliefs and expression suppression

The results showed that in Model 1, there was no significant indirect effect of emotion regulation beliefs on cross-cultural adjustment through smartphone addiction, with a mediated effect size of 0.048 (*p* > 0.05, 95% confidence interval [0.006,0.114]); there was a significant indirect effect of childhood emotion neglect on cross-cultural adjustment through emotion regulation beliefs, with a mediated effect the amount of mediated effect was −0.067 (*p* < 0.05, 95% confidence interval [−0.136, −0.014]); emotion regulation beliefs have no significant indirect effect on cross-cultural adjustment, through the expression suppression as well as the role of smartphone addiction, the mediated effect size was 0.016 (*p* > 0.05, 95% confidence interval [0.003, 0.037]); emotion regulation beliefs had a significant indirect effect on smartphone addiction through expression suppression, with a mediated effect size of −0.091 (*p* > 0.05, 95% confidence interval [−0.143, −0.053]).

### Mediation effects from childhood emotion neglect to cross-cultural adjustment through emotion regulation beliefs and cognitive appraisal

The results showed that in Model 2, cognitive reappraisal had a significant indirect effect on cross-cultural adjustment through smartphone addiction with a mediated effect size of −0.064 (*p* < 0.05, 95% confidence interval [−0.130, −0.027]); emotion regulation beliefs had a significant indirect effect on cross-cultural adjustment through smartphone addiction with a mediated effect size of 0.119 (*p* < 0.05, 95% confidence interval [0.056,0.205]); there was no significant indirect effect of childhood emotional neglect on cross-cultural adjustment through emotion regulation beliefs, with a mediated effect of −0.039 (*p* > 0.05, 95% confidence interval [−0.112, −0.005]); childhood emotional neglect affects smartphone addiction through emotion regulation beliefs, and there was no significant indirect effect of childhood emotional neglect on cross-cultural adjustment, the mediating effect size was −0.016 (*p* > 0.05, 95% confidence interval [−0.041, −0.004]).

## Discussion

This study examined the relationship between childhood emotional neglect, emotion regulation beliefs, emotional regulation strategies, smartphone addiction, and cross-cultural adjustment in a structural equation model. The results support the social cognitive theory that childhood emotional neglect has a significant negative effect on the cross-cultural adjustment and smartphone addiction through emotion regulation beliefs. Furthermore, two emotional regulation strategies demonstrated different patterns in the model. Emotion regulation beliefs affect smartphone addiction through the mediating role of expression suppression, and expression suppression has no significant effect on cross-cultural adjustment. On the contrast, emotion regulation beliefs have no significant effect on cognitive reappraisal, and cognitive reappraisal affects cross-cultural adjustment through the mediating role of smartphone addiction.

Childhood emotional neglect had a significant negative effect on both emotion regulation beliefs and cross-cultural adjustment, and emotion regulation beliefs had a significant positive effect on cross-cultural adjustment. Our findings fit with the social cognitive theory that early family environment influences individual cognition and behavior, and that cognition in turn influences individual behavior and adaptation to the environment. Social cognitive theory emphasizes that behavioral issues arises from irrational cognition which learned from earlier experiences, and that irrational cognition can affect an individual’s psychological wellbeing. An individual’s cognitive style is an important factor that influences social adaptation. According to learned helplessness research, individuals form certain beliefs from previous experiences (Seligman and Maier, 1967). Beliefs are related to attribution theory and learned helplessness (Dweck, 1975), and the way individuals attribute in childhood predicts behavior after experiencing frustration. When children attribute emotional neglect to uncontrollable factors, they experience feelings of helplessness and tend to believe that emotions are unregulated, which can have a negative impact on their future development (Dweck and Reppucci, 1973; Diener and Dweck, 1978). However, because of the inconsistent results of this pathway in the two model mediation tests, it cannot be concluded with certainty for the first hypothesis for the time being. Whether emotion regulation beliefs play a mediating role between childhood emotional neglect and cross-cultural adjustment, which will need to be further verified in future studies.

We found that the second hypothesis was partially supported; emotion regulation beliefs had a significant negative effect on both expression suppression and smartphone addiction, and expression suppression played a mediating role between emotion regulation beliefs and smartphone addiction. More specifically, individuals that endorse emotions cannot be regulated tend to use more expression suppression strategy and exposed themselves in a higher risk of smartphone addiction. This result is in line with some previous studies in which they pointed out that negative beliefs were a marker of addictive behavior (Spada et al., 2013; Hamonniere and Varescon, 2018). It is found that there is a positive association between negative beliefs about anxiety and problematic internet use (Marci et al., 2021), and that higher use of expression suppression by individuals significantly and positively predicts risk of smartphone addiction (Rozgonjuk and Elhai, 2021). As individuals tend to believe that (Mauss and Tamir, 2014), and thus tend to choose to suppress their emotional expression, while expression suppression leads to a decrease in positive emotional experiences (Brans et al., 2013). Smartphone applications provide a possible channel for these individuals to avoid emotional expression and obtain some entertaining experiences to improve their emotions.

Previous studies tend to suggest that emotion regulation beliefs predict increased use of cognitive reappraisals (De Castella et al., 2013), but our findings show no significant effect of emotion regulation beliefs on the cognitive reappraisal. This may be because individuals need to determine which emotion regulation strategy to use based on the available cognitive resources (Urry and Gross, 2010). If individuals endorse that they cannot use an emotion regulation strategy effectively (Gross, 2015), this may lead to an emotional regulation strategy outside of the “cognitive resource pool” being irrelevant to emotion regulation beliefs. This theory was also supported by Suri et al. (2015) experiment which noted that even though participants learned about using cognitive reappraisal, they often did not actively try to use this strategy, when individuals perceive that using cognitive reappraisal is difficult or that the costs of using cognitive reappraisal already outweigh the benefits it brings, then individuals do not choose to use cognitive reappraisals, even if they tend to believe that emotions can be regulated.

When we look at cross-cultural adjustment as one latent factor composed by both types of adaptation, no statistically significant association was found between expression suppression and cross-cultural adjustment, so the third hypothesis was not supported. In the current study, a two-factor correlation analysis found that expression suppression was significantly negatively correlated with psychological adaptation and insignificantly correlated with sociocultural adaptation, which is consistent with some of the previous studies conducted on Chinese populations (Soto et al., 2011; English and John, 2013; Zhao and Zhao, 2015). However, other research findings on the effects of expressive suppression have not reached the same conclusion, with scholars suggesting that expressive suppression may play a relatively positive function in collectivist cultures (Wei et al., 2013). As human societal values change from collectivism to individualism, individualistic values do not necessarily have positive consequences when they meet with Eastern cultural contexts (Wu et al., 2018). Thus, we suggest that cultural values may be a potential moderating variable in the relationship between expression inhibition and cross-cultural adaptation outcomes (Butler et al., 2007; Cheung and Park, 2010).

The data suggest that cognitive reappraisal has a significant positive effect on cross-cultural adjustment and smartphone addiction, smartphone addiction mediates between cognitive reappraisal and cross-cultural adjustment, and expression suppression has no significant effect on cross-cultural adjustment, so the fourth hypothesis was partially supported. Consistent with previous studies, frequent use of cognitive reappraisal is associated with higher cross-cultural adjustment, and cognitive reappraisal significantly reduces the risk of mental health problems in international students and promotes individual socio-cultural adjustment (Xv et al., 2022). In contrast to previous studies (Zhang and Jiang, 2017), we found that the more individuals tended to use cognitive reappraisals, the higher the level of smartphone addiction. This may be because cognitive reappraisal is not adaptive in all situations, and it is subject to situational variability and differences in individual coping preferences (Zhu et al., 2007; Haines et al., 2016). The cognitive reappraisal may rationalize the problematic smartphone use, leading to an increased risk of smartphone addiction and thus affecting individuals’ cross-cultural adjustment (Xie et al., 2019).

The results of the current study showed that 87.1% of Chinese students in Belarus are at high risk of mental health problems. The more international students tend to disbelieve that emotions can be regulated, the higher chance they were at the risk of smartphone addiction, lower levels of psychological adaptation and sociocultural adaptation. Positive emotion regulation beliefs among international students may help reduce problematic smartphone use and improve cross-cultural adjustment. This significant link from emotion regulation beliefs to cross-cultural adjustment through smartphone addiction could shed light on future prevention, and intervention for educators and policymakers. Specifically, to address the possibility that emotion can be regulated and education of useful regulating strategy could be of help. Further, to help international students build healthy connections with locals and to provide psychological support are crucial to preventing smartphone addiction and facilitating cross-cultural adjustment for international students.

There also are some shortcomings in this study. First, we used a cross-sectional research method that does not allow for causal inference and cannot exclude the possible effects of other additional variables; future research could combine experimental methods and longitudinal methods, to further explore the relationship between childhood maltreatment, emotion regulation, smartphone addiction, and cross-cultural adjustment. Second, we used a self-report survey to assess participants’ experiences of childhood emotional neglect, therefore were unable to accurately assess the type and extent of maltreatment experienced by participants during childhood; in the future, an observer reporting method could be used to further examine the relationship between childhood maltreatment and individual psychological health in adulthood. Again, this study only discusses the role of childhood emotional neglect, and future research could explore the effects of childhood physical maltreatment and neglect, emotional maltreatment, and sexual abuse on individuals’ emotion beliefs and cross-cultural adjustment. In addition, we were unable to obtain information on type of the smartphone use as well as the specific duration of use from self-reported smartphone addiction assessments, and therefore could not accurately estimate the degree of smartphone addiction among participants; future research could incorporate complementary tools such as smartphone software to specifically explore the relationship between different smartphone use patterns and cross-cultural adjustment. Finally, current research on emotion beliefs focuses on two basic types of beliefs: beliefs about the controllability of emotions, and beliefs about the usefulness of emotions, but these are not the only types of beliefs about emotions that people can hold (Ben-Artzi et al., 1995). These two emotion beliefs may change when the specific attributes of the emotion, the situation under discussion, are different (Ford and Gross, 2018). In light of this, in the future we can explore the mechanisms by which various emotion beliefs act on individuals’ cross-cultural adjustment in different situations, we can also combine experimental manipulation and clinical interventions to elucidate the causal role of different emotion beliefs in clinical treatments (e.g., addictive disorders) (De Castella et al., 2015; Predatu et al., 2020).

## Conclusion

As the unstable regional security situation and the wide spread of COVID-19 may negatively affect the cross-cultural adjustment of Chinese students in Belarus, this study constructs a possible model framework to provide a solution for the cross-cultural adjustment of this group. The results of this study indicate that childhood emotional neglect has a significant negative effect on both emotion regulation beliefs and cross-cultural adjustment. Childhood emotional neglect was found associated with cross-cultural adjustment and smartphone addiction through the path from emotion regulation beliefs and emotional regulation strategy expression suppression. The other regulation strategy cognitive reappraisal affects cross-cultural adjustment through the mediation of smartphone addiction. Researchers are encouraged to further explore the role of different emotional beliefs in relation to emotion regulation strategies, smartphone addiction and cross-cultural adjustment when the specific attributes of the emotion, and the situation under discussion, are different. In summary, this study deepens the understanding of the impact of childhood maltreatment on the long-term development of individuals; it has implications for preventing smartphone addiction and promoting cross-cultural adjustment of international students.

## Data availability statement

The raw data supporting the conclusions of this article will be made available by the authors, without undue reservation.

## Ethics statement

The studies involving human participants were reviewed and approved by Ethics Committee of the Belarusian State University. The patients/participants provided their written informed consent to participate in this study.

## Author contributions

HW and LF contributed to the data analysis. HW and AS contributed to the data interpretation. All authors conceived the assessment, drafted the manuscript, critically revised the manuscript, and approved the final version for publication.

## Footnotes

1
https://www.wjx.cn/

## References

[B1] Arnett J. J. Žukauskienė R. Sugimura K. (2014). The new life stage of emerging adulthood at ages 18–29 years: Implications for mental health. *Lancet Psychiatry* 1 569–576. 10.1016/S2215-0366(14)00080-7 26361316

[B2] Bandura A. (1997). *Self-efficacy: The exercise of control.* New York: W H Freeman/Times Books/Henry Holt & Co.

[B3] Ben-Artzi E. Mikulincer M. Glaubman H. (1995). The multifaceted nature of self-consciousness: Conceptualization, measurement, and consequences. *Imag. Cogn. Pers.* 15 17–43. 10.2190/AV07-Y0K1-8D71-FBAA 22612255

[B4] Bender M. van Osch Y. Sleegers W. Ye M. (2019). Social support benefits psychological adaptation of international students: Evidence from a meta-analysis. *J. Cross-Cult. Psychol.* 50 827–847. 10.1177/0022022119861151

[B5] Bernstein D. Fink L. Bernstein D. P. (1998). Childhood trauma questionnaire: A retrospective self-report manual. *Add. Behav.* 23 855–868. 10.1016/S0306-4603(98)00072-0

[B6] Brans K. Koval P. Verduyn P. Lim Y. L. Kuppens P. (2013). The regulation of negative and positive affect in daily life. *Emotion* 13 926–939. 10.1037/a0032400 23731436

[B7] Butler E. A. Lee T. L. Gross J. J. (2007). Emotion regulation and culture: Are the social consequences of emotion suppression culture-specific? *Emotion* 7 30–48. 10.1037/1528-3542.7.1.30 17352561

[B8] Byrne B. M. (1998). *Structural equation modeling with LISREL, PRELIS, and SIMPLIS: Basic concepts, applications, and programming.* Hillsdale, NJ: Lawrence Erlbaum Associates Publishers.

[B9] Cheung R. Y. Park I. J. (2010). Anger suppression, interdependent self-construal, and depression among Asian American and European American college students. *Cultur. Divers. Ethnic. Minor. Psychol.* 16 517–525. 10.1037/a0020655 21058815 PMC3058745

[B10] Cohen J. R. Menon S. V. Shorey R. C. Le V. D. Temple J. R. (2017). The distal consequences of physical and emotional neglect in emerging adults: A person-centered, multi-wave, longitudinal study. *Child Abuse Neglect* 63 151–161. 10.1016/j.chiabu.2016.11.030 27923183 PMC5282706

[B11] De Castella K. Goldin P. Jazaieri H. Heimberg R. G. Dweck C. S. Gross J. J. (2015). Emotion beliefs and cognitive behavioural therapy for social anxiety disorder. *Cogn. Behav. Ther.* 44 128–141. 10.1080/16506073.2014.974665 25380179

[B12] De Castella K. Goldin P. Jazaieri H. Ziv M. Dweck C. S. Gross J. J. (2013). Beliefs about emotion: Links to emotion regulation, well-being, and psychological distress. *Basic Appl. Soc. Psychol.* 35 497–505. 10.1080/01973533.2013.840632

[B13] De Castella K. Goldin P. Jazaieri H. Ziv M. Heimberg R. G. Gross J. J. (2014). Emotion beliefs in social anxiety disorder: Associations with stress, anxiety, and well-being. *Austr. J. Psychol.* 66 139–148. 10.1111/ajpy.12053

[B14] Diener C. I. Dweck C. S. (1978). An analysis of learned helplessness: Continuous changes in performance, strategy, and achievement cognitions following failure. *J. Pers. Soc. Psychol.* 36 451–462. 10.1037/0022-3514.36.5.451

[B15] Dweck C. S. (1975). The role of expectations and attributions in the alleviation of learned helplessness. *J. Pers. Soc. Psychol.* 31 674–685. 10.1037/h0077149

[B16] Dweck C. S. Reppucci N. D. (1973). Learned helplessness and reinforcement responsibility in children. *J. Pers. Soc. Psychol.* 25 109–116. 10.1037/h0034248

[B17] Elhai J. D. Dvorak R. D. Levine J. C. Hall B. J. (2017). Problematic smartphone use: A conceptual overview and systematic review of relations with anxiety and depression psychopathology. *J. Affect. Disord.* 207 251–259. 10.1016/j.jad.2016.08.030 27736736

[B18] English T. John O. P. (2013). Understanding the social effects of emotion regulation: The mediating role of authenticity for individual differences in suppression. *Emotion* 13 314–329. 10.1037/a0029847 23046456

[B19] Ford B. Q. Gross J. J. (2018). Emotion regulation: Why beliefs matter. *Can. Psychol.* 59 1–14. 10.1037/cap0000142

[B20] Ford B. Q. Feinberg M. Lam P. Mauss I. B. John O. P. (2019). Using reappraisal to regulate negative emotion after the 2016 US Presidential election: Does emotion regulation trump political action? *J. Pers. Soc. Psychol.* 117 998–1015. 10.1037/pspp0000200 29952576

[B21] Ford B. Q. Lwi S. J. Gentzler A. L. Hankin B. Mauss I. B. (2018). The cost of believing emotions are uncontrollable: Youths’ beliefs about emotion predict emotion regulation and depressive symptoms. *J. Exp. Psychol. Gen.* 147 1170–1190. 10.1037/xge0000396 29620380

[B22] Fortes A. B. Broilo P. L. Lisboa C. S. D. M. (2021). Smartphone use and psychological well-being: The moderating role of emotion regulation. *Trends Psychol.* 29 189–203. 10.1007/s43076-020-00051-1

[B23] Frodl T. Reinhold E. Koutsouleris N. Reiser M. Meisenzahl E. M. (2010). Interaction of childhood stress with hippocampus and prefrontal cortex volume reduction in major depression. *J. Psychiatr. Res.* 44 799–807. 10.1016/j.jpsychires.2010.01.006 20122698

[B24] Gilbert R. Widom C. S. Browne K. Fergusson D. Webb E. Janson S. (2009). Burden and consequences of child maltreatment in high-income countries. *Lancet* 373 68–81. 10.1016/S0140-6736(08)61706-7 19056114

[B25] Goldberg D. P. (1978). *Manual of the General Health Questionnaire.* Windsor: NFER Publishing Company.

[B26] Gross J. J. (1998). Antecedent-and response-focused emotion regulation: Divergent consequences for experience, expression, and physiology. *J. Pers. Soc. Psychol.* 74 224–237. 10.1037/0022-3514.74.1.224 9457784

[B27] Gross J. J. (2015). Emotion regulation: Current status and future prospects. *Psychol. Inq.* 26 1–26. 10.1080/1047840X.2014.940781

[B28] Gross J. J. John O. P. (2003). Individual differences in two emotion regulation processes: Implications for affect, relationships, and well-being. *J. Pers. Soc. Psychol.* 85 348–362. 10.1037/0022-3514.85.2.348 12916575

[B29] Gulbro R. D. Herbig P. (1999). Cultural differences encountered by firms when negotiating internationally. *Indus. Manage. Data Syst.* 99 47–53. 10.1108/02635579910261059

[B30] Haines S. J. Gleeson J. Kuppens P. Hollenstein T. Ciarrochi J. Labuschagne I. (2016). The wisdom to know the difference: Strategy-situation fit in emotion regulation in daily life is associated with well-being. *Psychol. Sci.* 27 1651–1659. 10.1177/0956797616669086 27738099

[B31] Hamonniere T. Varescon I. (2018). Metacognitive beliefs in addictive behaviours: A systematic review. *Add. Behav.* 85 51–63. 10.1016/j.addbeh.2018.05.018 29852356

[B32] Hu L. T. Bentler P. M. (1999). Cutoff criteria for fit indexes in covariance structure analysis: Conventional criteria versus new alternatives. *Structural Equ. Model.* 6 1–55. 10.1080/10705519909540118

[B33] Hu S. Liu H. Zhang S. Wang G. (2020). Proactive personality and cross-cultural adjustment: Roles of social media usage and cultural intelligence. *Int. J. Intercult. Relations* 74 42–57. 10.1016/j.ijintrel.2019.10.002

[B34] Jain S. C. (ed.) (2006). *Emerging economies and the transformation of international business: Brazil, Russia, India and China (BRICs).* Cheltenham, UK: Edward Elgar Publishing. 10.4337/9781847202987

[B35] Ju C. E. (2011). Intercultural adaptation to Russia of students from Asia, Africa, Latin America and the Middle East . *RUDN J. Psychol. Pedag.* 3 6–11.

[B36] Kim S. E. Kim J. W. Jee Y. S. (2015). Relationship between smartphone addiction and physical activity in Chinese international students in Korea. *J. Behav. Add.* 4 200–205. 10.1556/2006.4.2015.028 26551911 PMC4627682

[B37] Konstantinov V. Gritsenko V. Reznik A. Isralowitz R. (2022). The impact of covid-19 on health and well-being: Foreign medical students in Eastern Europe. *Soc. Sci.* 11:393. 10.3390/socsci11090393

[B38] Kurapov A. Pavlenko V. Drozdov A. Bezliudna V. Reznik A. Isralowitz R. (2022). Toward an understanding of the Russian-Ukrainian war impact on university students and personnel. *J. Loss Trauma* 27 1–8. 10.1080/15325024.2022.2084838

[B39] Leung L. (2008). Linking psychological attributes to addiction and improper use of the mobile phone among adolescents in Hong Kong. *J. Child. Media* 2 93–113. 10.1080/17482790802078565

[B40] Liu S. S. Ling W. Q. (2009). Multiple mediation models and their applications. *Psychol. Sci.* 32, 433–435,407.

[B41] MacCallum R. C. Browne M. W. Sugawara H. M. (1996). Power analysis and determination of sample size for covariance structure modeling. *Psychol. Methods* 1 130–149. 10.1037/1082-989X.1.2.130

[B42] Maheu F. S. Dozier M. Guyer A. E. Mandell D. Peloso E. Poeth K. (2010). A preliminary study of medial temporal lobe function in youths with a history of caregiver deprivation and emotional neglect. *Cogn. Affect. Behav. Neurosci.* 10 34–49. 10.3758/CABN.10.1.34 20233954 PMC2926942

[B43] Marci T. Marino C. Sacchi C. Lan X. Spada M. M. (2021). Problematic Internet Use in early adolescence: The role of attachment and negative beliefs about worry. *J. Behav. Add.* 10 194–200. 10.1556/2006.2021.00001 33475528 PMC8969852

[B44] Matsumoto D. Yoo S. H. LeRoux J. A. (2007). Emotion and intercultural adjustment. *Handb. Appl. Linguistics* 7 77–97. 10.1515/9783110198584.1.77

[B45] Mauss I. B. Tamir M. (2014). *Emotion goals: How their content, structure, and operation shape emotion regulation.* New York: The Guilford Press.

[B46] Ministry of Education of the Republic of Belarus (2022). *Three decades of Belarusian-Chinese relations.* Available online at: https://edu.gov.by/by-be/news/tri-desyatiletiya-belorusskokitayskikh-otnosheniy/?sphrase_id=262641 (accessed on April 15, 2022).

[B47] Ministry of Science and Higher Education of the Russian Federation (2022). *Higher education.* Available online at: https://minobrnauki.gov.ru/action/stat/highed/ (accessed on April 15, 2022).

[B48] Moe.gov.cn (2022). *Statistics of 2019 Study Abroad - Government Portal of the Ministry of Education of the People’s Republic of China.* Available online at: http://www.moe.gov.cn/jyb_xwfb/gzdt_gzdt/s5987/202012/t20201214_505447.html (accessed on April 15, 2022)

[B49] Müller L. E. Bertsch K. Bülau K. Herpertz S. C. Buchheim A. (2019). Emotional neglect in childhood shapes social dysfunctioning in adults by influencing the oxytocin and the attachment system: Results from a population-based study. *Int. J. Psychophysiol.* 136 73–80. 10.1016/j.ijpsycho.2018.05.011 29859994

[B50] Popescu M. L. Drumm R. Dewan S. Rusu C. (2010). Childhood victimization and its impact on coping behaviors for victims of intimate partner violence. *J. Fam. Viol.* 25 575–585. 10.1007/s10896-010-9317-5

[B51] Predatu R. David D. O. Maffei A. (2020). Beliefs about emotions, negative meta-emotions, and perceived emotional control during an emotionally salient situation in individuals with emotional disorders. *Cogn. Ther. Res.* 44 287–299. 10.1007/s10608-019-10064-5

[B52] Reuters (2022). *FEATURE-Foreign students fleeing Ukraine, battle racism, extortion*. Available online at: https://www.reuters.com/article/ukraine-discrimination-students-idAFL8N2V48CH (accessed April 15, 2022).

[B53] Romero C. Master A. Paunesku D. Dweck C. S. Gross J. J. (2014). Academic and emotional functioning in middle school: The role of implicit theories. *Emotion* 14 227–234. 10.1037/a0035490 24512251

[B54] Rozgonjuk D. Elhai J. D. (2021). Emotion regulation in relation to smartphone use: Process smartphone use mediates the association between expressive suppression and problematic smartphone use. *Curr. Psychol.* 40 3246–3255. 10.1007/s12144-019-00271-4

[B55] Rozgonjuk D. Levine J. C. Hall B. J. Elhai J. D. (2018). The association between problematic smartphone use, depression and anxiety symptom severity, and objectively measured smartphone use over one week. *Comp. Hum. Behav.* 87 10–17. 10.1016/j.chb.2018.05.019

[B56] Salokangas R. K. Schultze-Lutter F. Schmidt S. J. Pesonen H. Luutonen S. Patterson P. (2019). Childhood physical abuse and emotional neglect are specifically associated with adult mental disorders. *J. Mental Health* 29 1–9. 10.1080/09638237.2018.1521940 30675805

[B57] Schroder H. S. Dawood S. Yalch M. M. Donnellan M. B. Moser J. S. (2015). The role of implicit theories in psychological health symptoms, emotion regulation, and hypothetical treatment choices in college students. *Cogn. Ther. Res.* 39 120–139. 10.1007/s10608-014-9652-6 35474696 PMC9037854

[B58] Searle W. Ward C. (1990). The prediction of psychological and sociocultural adjustment during cross-cultural transitions. *Int. J. Intercult. Relations* 14 449–464. 10.1016/0147-1767(90)90030-Z

[B59] Seligman M. E. Maier S. F. (1967). Failure to escape traumatic shock. *J. Experipsychol. Psychol.* 74 1–9. 10.1037/h0024514 6032570

[B60] Shi J. (2019). *Study on the Status Quo of Intercultural Psychological Adaptation of Chinese Students in Kyrgyzstan.* (Ph.D.thesis). China: Xinjiang Normal University.

[B61] Soto J. A. Perez C. R. Kim Y. H. Lee E. A. Minnick M. R. (2011). Is expressive suppression always associated with poorer psychological functioning?A cross-cultural comparison between European Americans and Hong Kong Chinese. *Emotion* 11 1450–1455. 10.1037/a0023340 21707152

[B62] Spada M. M. Caselli G. (2017). The metacognitions about online gaming scale: Development and psychometric properties. *Add. Behav.* 64 281–286. 10.1016/j.addbeh.2015.07.007 26210288

[B63] Spada M. M. Caselli G. Wells A. (2013). A triphasic metacognitive formulation of problem drinking. *Clin. Psychol. Psychother.* 20 494–500. 10.1002/cpp.1791 22589026

[B64] Stoltenborgh M. Bakermans-Kranenburg M. J. Alink L. R. van IJzendoorn M. H. (2015). The prevalence of child maltreatment across the globe: Review of a series of meta-analyses. *Child Abuse Rev.* 24 37–50. 10.1002/car.2353

[B65] Sun Y. Nolan C. (2021). Emotion regulation strategies and stress in Irish college students and Chinese international college students in Ireland. *J. Int. Students* 11 853–873. 10.32674/jis.v11i4.2516

[B66] Sun Y. Bo S. Y. Lv J. J. (2020). Brain network analysis of cognitive reappraisal and expressive suppression strategies: Evidence from EEG and ERP. *Acta Psychol. Sin.* 52 12–25. 10.3724/SP.J.1041.2020.00012

[B67] Suri G. Whittaker K. Gross J. J. (2015). Launching reappraisal: It’s less common than you might think. *Emotion* 15 73–77. 10.1037/emo0000011 24999912

[B68] Tamir M. John O. P. Srivastava S. Gross J. J. (2007). Implicit theories of emotion: Affective and social outcomes across a major life transition. *J. Pers. Soc. Psychol.* 92 731–744. 10.1037/0022-3514.92.4.731 17469955

[B69] Tao Y. (2012). *The Intercultural Adjustment of Chinese Interns in The United States:a case of disney international program participants in orlando.* (Ph.D.thesis). China: Shanghai International Studies University.

[B70] Trumello C. Babore A. Candelori C. Morelli M. Bianchi D. (2018). Relationship with parents, emotion regulation, and callous-unemotional traits in adolescents’internet addiction. *BioMed Res. Int.* 2018:7914261. 10.1155/2018/7914261 29951544 PMC5989287

[B71] Tu Y. T. Lin S. Y. Chang Y. Y. (2011). A cross-cultural comparison by individualism/collectivism among Brazil, Russia, India and China. *Int. Bus. Res.* 4 175–182. 10.5539/ibr.v4n2p175

[B72] Urry H. L. Gross J. J. (2010). Emotion regulation in older age. *Curr. Direct. Psychol. Sci.* 19 352–357. 10.1177/0963721410388395

[B73] Veilleux J. C. Chamberlain K. D. Baker D. E. Warner E. A. (2021). Disentangling beliefs about emotions from emotion schemas. *J. Clin. Psychol.* 77 1068–1089. 10.1002/jclp.23098 33319397

[B74] Veilleux J. C. Salomaa A. C. Shaver J. A. Zielinski M. J. Pollert G. A. (2015). Multidimensional assessment of beliefs about emotion:Development and validation of the emotion and regulation beliefs scale. *Assessment* 22 86–100. 10.1177/1073191114534883 24835246

[B75] Wang K. T. Heppner P. P. Wang L. Zhu F. (2015). Cultural intelligence trajectories in new international students: Implications for the development of cross-cultural competence. *Int. Perspect. Psychol.* 4 51–65. 10.1037/ipp0000027

[B76] Wang L. Lu Y. P. Li Z. Q. (2007). Test of Emotion Regulation Scale in adolescents. *Chin. J. Clin. Psychol.* 15 236–238.

[B77] Wang W. J. (2016). *The Relationship between Implicit Beliefs of Emotion and Emotion, Emotion Regulation: Behavior and Physical Evidence.* (Ph.D.thesis). Yinchuan, China: Ningxia University.

[B78] Wang Y. W. Xiang J. M. Yang K. R. Yang Z. H. Wu C. X. (2019). Influence of childhood maltreatment on adult psychology and social behavior. *Chin. J. School Doctor* 33 738–739.

[B79] Ward C. Kennedy A. (1992). Locus of control, mood disturbance, and social difficulty during cross-cultural transitions. *Int. J. Intercult. Relations* 16 175–194. 10.1016/0147-1767(92)90017-O

[B80] Ward C. Kennedy A. (1994). Acculturation strategies, psychological adjustment, and sociocultural competence during cross-cultural transitions. *Int. J. Intercult. Relations* 18 329–343. 10.1016/0147-1767(94)90036-1

[B81] Ward C. Kennedy A. (1999). The measurement of sociocultural adaptation. *Int. J. Intercult. Relations* 23 659–677. 10.1016/S0147-1767(99)00014-0

[B82] Ward C. Rana-Deuba A. (1999). Acculturation and adaptation revisited. *J. Cross-Cult. Psychol.* 30 422–442. 10.1177/0022022199030004003

[B83] Waters S. F. Thompson R. A. (2014). Children’s perceptions of the effectiveness of strategies for regulating anger and sadness. *Int. J. Behav. Develop.* 38 174–181. 10.1177/0165025413515410

[B84] Wei M. Su J. C. Carrera S. Lin S. P. Yi F. (2013). Suppression and interpersonal harmony: A cross-cultural comparison between Chinese and European Americans. *J. Counsel. Psychol.* 60 625–633. 10.1037/a0033413 23978268

[B85] Wheaton B. Muthen B. Alwin D. F. Summers G. F. (1977). Assessing reliability and stability in panel models. *Sociol. Methodol.* 8 84–136. 10.2307/270754

[B86] Womersley J. S. Hemmings S. M. J. Ziegler C. Gutridge A. Ahmed-Leitao F. Rosenstein D. (2020). Childhood emotional neglect and oxytocin receptor variants: Association with limbic brain volumes. *World J. Biol. Psychiatry* 21 513–528. 10.1080/15622975.2019.1584331 30806136

[B87] Wu M. S. Zhou C. Chen H. Cai H. Sundararajan L. (2018). Cultural value mismatch in urbanizing China: A large-scale analysis of collectivism and happiness based on social media and nationwide survey. *Int. J. Psychol.* 53 54–63. 10.1002/ijop.12523 30239987

[B88] Wu Q. Luo J. Bai J. Hou M. Li X. (2019). Effect of security on mobile addiction: Mediating role of actual social avoidance. *Psychol. Dev. Educ.* 35 589–596.

[B89] Xie L. L. Ji Y. Li C. Y. Hou Z. H. Liu Y. H. (2019). The status of “phubbing” and its relationship with social adjustment in college students. *China J. Health Psychol.* 27 256–260.

[B90] Xiong J. Zhou Z. K. Chen W. You Z. Q. Zhai Z. Y. (2012). Development of the Mobile Phone Addiction Tendency Scale for College Students. *Chin. Psychol. Health J.* 26 222–225. 10.1037/t74211-000

[B91] Xv Y. Y. Zhang X. Y. Yang J. (2022). Mediating role of emotional regulation strategy in the influence of social support on psychological health of overseas students in China during COVID-19 epidemic. *China J. Health Psychol.* 30 452–457.

[B92] Yang T. Z. Huang L. Wu Z. Y. (2003). The application of Chinese health questionnaire for psychological disorder screening in community settings in mainland China. *Chin. J. Epidemiol.* 24 769–773. 14521766

[B93] Ye B. J. Fang X. T. Yang Q. Zheng Q. Liu L. L. Gou S. Y. (2017). The effects of difficulties in emotional regulation on college students’ mobile phone addiction: The chain mediating effect of facial negative physical self and social avoidance and distress. *Psychol. Dev. Educ.* 33 249–256.

[B94] Young J. E. Klosko J. S. Weishaar M. E. (2003). *Schema therapy.* New York: Guilford, 254.

[B95] Yu G. L. Li S. Zhao F. Q. (2020). Childhood maltreatment and prosocial behavior among Chinese adolescents:Roles of empathy and gratitude. *Child Abuse Neglect* 101:104319. 10.1016/j.chiabu.2019.104319 31954947

[B96] Zhang J. Z. Jiang Y. (2017). Effect of college students” emotion regulation strategies on interpersonal disturbances and mobile phone addiction. *Modern Prevent. Med.* 44 3356–3359.

[B97] Zhang L. L. (2018). *The Chinese Translation and Application of the Emotion and Regulation Beliefs Scale for Nursing Students.* (Ph.D.thesis). China: Jinzhou Medical University.

[B98] Zhang M. (2011). Reliability and validity of the Chinese version of CTQ-SF. *Chin. J. Pub. Health* 27 669–670.

[B99] Zhao X. Zhang B. R. Zhang P. Pan L. Zhou R. L. (2015). Reliability and validity of emotion regulation questionnaire in middle school students. *Chin. J. Clin. Psychol.* 23 22–25.

[B100] Zhao Y. Zhao G. (2015). Emotion regulation and depressive symptoms: Examining the mediation effects of school connectedness in Chinese late adolescents. *J. Adolesc.* 40 14–23. 10.1016/j.adolescence.2014.12.009 25600512

[B101] Zhou H. Long L. (2004). Statistical remedies for common method biases. *Adv. Psychol. Sci.* 12 942–950.

[B102] Zhu X. Z. Luo F. S. Yao S. Q. Randy P. A. John R. Z. A. (2007). Reliability and validity of the cognitive emotion regulation questionnaire—Chinese version. *Chin. J. Clin. Psychol.* 15 121–124.

