# Supplementary material for: Smartphone addiction and cross-cultural adjustment among overseas Chinese students: The role of emotion regulation beliefs and strategies
Source: Front Psychol. 2022 Oct 10;13:1009347. doi: 10.3389/fpsyg.2022.1009347 (PMC9590311; doi:10.3389/fpsyg.2022.1009347)
Supplement: Supplementary file 1 [file Table_1.docx]

**1. Demographics Questionnaire人口统计学调查问卷**

**Instructions:** this part contains basic personal information. Please read the following information carefully and choose the appropriate option according to your actual situation. 该部分是对于个人基本信息的调查,请您仔细阅读以下内容,并根据您的实际情况,选择相应的选项

**1. Gender** [single choice]* 性别 [单选题]

○male 男

○female 女

**2. Age** [fill in the blank]* 年龄 [填空题]

-----------------

**3. The only child** [single choice]* 独生 [单选题]

○Yes 是

○No 否

**4. Parents' marital status** [single choice]* 父母的婚姻状况 [单选题]

○Parents living together 父母共同生活

○Parents divorced 父母离异

**5. Do you think your family's living standard belongs to** [single-choice question] **among your classmates*** 你觉得你家的生活水平在同学当中属于 [单选题]

○ poor 贫困

○ not too rich 不太富裕

○ average 一般

○ rich 富裕

**6. Education level** [single choice]* 受教育水平 [单选题]

○Below Bachelor's degree 本科以下

○Undergraduate 本科

○Master and above 硕士及以上

**7. Please choose an adjective that matches your academic performance** [multiple choice]* 请选择一个与您学业成绩相符合的形容词 [单选题]

○ poor 较差

○ fair 一般

○ good 良好

○ excellent 优秀

**8. Living time in Belarus [single choice] *** 在白俄罗斯生活时间 [单选题]

○Under 6 months ６个月以下

○6~12 months ６~12个月

○1~2 years 1~2年

○2~3 years 2~3年

○3 years or more 3年以上

**2. 12-item General Health Questionnaire 12项一般健康调查问卷**

**Instructions:** Please choose an option that best represents your psychological state according to your feelings in the past month. 请根据您最近一个月来的感受,选择一个最能代表自己心理状态的选项

- Not at all 一点也没有
- As much as usual 和以前一样多
- More serious than usual 比以前多
- Much more serious than usual 比以前多很多

**Items 项目**

**1. I feel unable to overcome my difficulties** 我感到不能克服自己的困难

**2. For some things I can make my own decisions** 对一些事情能由我自己做决定

**3. I feel unhappy and depressed** 我感到不快乐和压抑

**4. I feel like I am doing something meaningful** 我感到自己在做有意义的事情

**5. I can concentrate no matter what I do** 不论做什么我都能集中精力

**6. I suffer from insomnia due to excessive worry** 我因过度担心而失眠

**7. I feel like a worthless person** 我感到自己是一个一无是处的人

**8. Overall I feel happy** 从总体上说我觉得快乐

**9. I am able to enjoy my normal daily activities** 我能够享受每天正常的活动

**10. I am able to face my own problems** 我能够正视自己的问题

**11. I felt myself in tension** 我感到自己处于紧张中

**12. I lost confidence in myself** 我对自己丧失信心

*The reliability of the Chinese version of the GHQ-12 has been confirmed (Yang, Huang & Wu, 2003). The Cronbach's Alpha coefficient for this scale was 0.792.*

**3. Childhood Trauma Questionnaire-Short Form童年创伤调查表--简表**

**Instruction:** Please choose an option that best represents situation according to your childhood (before 16 years old) growth experience. 请根据您童年期（16岁以前）的成长经历,选择一个最能代表自己情况的选项

- Never 从不
- Occasionally 偶尔
- Sometimes 有时
- Often 常常
- Always 总是

**Items项目**

**1. Someone in my family makes me feel important or unusual** 家里有人使我觉得自己很重要或不一般

**2. Some people in my family call me "idiot", "slacker" or "ugly"** 家里有人喊我“笨蛋”“懒虫”或“丑八怪”等

**3. I feel that my family loves me** 我感到家里人爱我

**4. I feel like my parents wish they had never given birth to me** 我觉得父母希望从来没有生过我

**5. Family members care about each other** 家里人彼此互相关心

**6. I feel like someone in my family hates me** 我觉得家里有人憎恨我

**7. The family is very close** 家里人关系很亲密

**8. Someone in my family said mean or insulting things to me** 家里有人向我说过刻薄或侮辱性的话

**9. Families are my source of strength and support** 家是我力量和支持的源泉

**10. I thought I was emotionally abused** 我认为我受到了情感虐待

*The Chinese version of the CTQ-SF has reliable letter validity in a sample of Chinese university students (Zhang, 2011). The Cronbach's Alpha coefficient for the emotional neglect dimension was 0.791*

**4. Emotion and Regulation Beliefs Scale 情绪调控信念量表**

**Instructions:** The following are some descriptions related to your emotions. Please choose an option that best represents your own views according to your actual situation. 下面是一些与您的情绪有关的描述,请根据您的实际情况,选择一个最能代表自己看法的选项

- Strongly Disagree 非常不同意
- Disagree 不同意
- Fair/Ok 普通/还好
- Agree 同意
- Strongly Agree 非常同意

**Items 项目**

**1. People are able to learn to control or limit their emotions** 人们能够学习控制或限制自己的情绪

2. **People are swayed by their emotions** 人们被自己的情绪所左右

**3. The process of trying to improve your mood is a worthwhile experience** 努力改善情绪的过程是很值得的一种经历

**4. When people feel down, they have to wait until they are in a good mood before they can reach the previous level of work efficiency** 当人们感觉情绪低落时,必须等到情绪好的时候才能达到之前工作效率水平

**5. It would be better if people knew where their emotions came from** 人们如果能知道自己情绪的出处就更好了

**6. When an emotion arises, it continues until the surrounding environment changes** 当情绪产生时，它会不断持续直到周围的环境发生了变化

**7. When people express their emotions, they are completely controlled by them** 当人们表露自己的情绪的时候，他们完全被这种情绪所控制

**8. Learning how to change strong emotions is a worthwhile pursuit** 学习如何改变强烈的情绪是一个值得追求的事

**9. Regardless of the circumstances, it is possible to change strong emotions through effort** 无论在何种情况下，通过努力改变强烈的情绪都是可能的

**10. When people feel angry, they may actually take out that anger on people or things around them** 当人们感觉愤怒时，实际上可能会把这种愤怒发泄在周围人或物上

**11. It would be better if people spent more time learning how to control their emotions** 人们如果能花费更多的时间去学习如何控制自己的情绪就更好了

**12. Strong emotions cause people to do things they wouldn't normally do** 强烈的情绪会使人做平常不会做的事情

**13. When grief takes over one's emotions, he/she cannot do anything but wallow in pain** 当悲伤占据一个人的情感时，一个人除了沉溺在痛苦之中，将无法做任何事

**14. People benefit from how they control their emotions** 人们从如何控制自己的情绪中获益

**15. People actually have a hard time acting against their emotions** 人们实际上很难违背自己的感情行事

**16. Emotions make people out of control** 情绪让人失控

*Zhang adapted the ERBS to suit Chinese culture deleting three entries with a correlation coefficient of less than 0.30 with the total score (Zhang, 2018). The Cronbach's Alpha coefficient for the scale was 0.861.*

1. **Mobile Phone Addiction Tendency Scale for College Students**

**大学生手机成瘾倾向量表**

*Information about this scale presented in Supplementary Form 2*

**6. Emotion Regulation Questionnaire 情绪调节量表**

**Instructions:** Below you will read some descriptions of your emotional life. Please choose an option that is consistent with your actual situation. 以下是一些对您情绪生活有关的描述,请选择一个与您实际情况相符合的选项

- Strongly Disagree 非常不赞同
- Disagree 很不赞同
- Somewhat Disagree 稍不赞同
- Neither Agree or Disagree 不确定
- Somewhat Agree 稍赞同
- Agree 很赞同
- Strongly Agree 非常赞同

**Items 项目**

**1. When I want to feel some positive emotion (like happiness or joy), I change the way I think about it** 当我想感受一些积极的情绪（如快乐或高兴）时，我会改变自己思考问题的角度

**2. I do not express my emotions** 我不会表露自己的情绪

**3. When I want to feel less negative emotions (such as sadness or anger), I change the way I think about things** 当我想少感受一些消极的情绪 （如悲伤或愤怒） 时，我会改变自己思考问题的角度

**4. When positive emotions are felt, I am careful not to let them show** 当感受到积极情绪时，我会很小心地不让它们表露出来

**5. When I faced with a stressful situation, I allow myself to think about it in a way that helps to maintain calm** 在面对压力情境时，我会使自己以一种有助于保持平静的方式来考虑它

**6. The way I control my emotions is not to express them** 我控制自己情绪的方式是不表达它们

**7. When I want to feel more positive emotions, I change the way I think about the situation** 当我想多感受一些积极的情绪时，我会改变自己对情境的考虑方式

**8. I control my emotions by changing the way I think about the situation** 我会通过改变对情境的考虑方式来控制自己的情绪

**9. When negative emotions are felt, I am sure not to express them** 当感受到消极的情绪时，我确定不会表露它们

**10. When I want to feel less negative emotions, I change the way I think about the situation** 当我想少感受一些消极的情绪时，我会改变自己对情境的考虑方式

*The reliability and validity of the Chinese version of this scale were confirmed to be at a reasonable level (Zhao et al., 2015). The Cronbach's alpha coefficients for the two dimensions of the questionnaire were 0.868 and 0.733, respectively, and the total Cronbach's alpha coefficient was 0.851.*

**7. Sociocultural Adaptation Scale 社会文化适应量表**

**Instructions:** Below you will read some descriptions of your life in Belarus. Please choose an option based on how well it corresponds with your experience. 以下是一些对您在白俄罗斯生活有关的描述,根据与您经历的符合程度选择一个选项

- Extremely difficult 极其困难
- Fairly difficult 相当困难
- Moderately difficult 中等难度
- Some difficulty 有一点困难
- No difficulty 没有困难

**Items 项目**

**1. Make friends** 交朋友

**2. Using the transportation system** 使用交通系统

**3. Make yourself understood** 使自己得到理解

**4. Get used to the rhythm of life** 习惯生活节奏

**5. Shopping** 购物

**6. Participate in social events and gatherings** 参加社会活动/聚会/盛大的集会

**7. Dealing with unpleasant people** 与令人不偷快的人打交道

**8. Get used to local food** 习惯当地食物

**9. Follow the prescribed rules** 遵守规定规则

**10. Dealing with powerful people** 与有权人士打交道

**11. Adapt to local accommodation** 适应当地住宿

**12. Coping with unsatisfactory service** 应付不令人满意的服务

**13. Cope with the climate** 应付气候

**14. Understand the Belarusian cultural system** 理解白俄罗斯文化体系

**15. Understand ethnic or cultural differences of Belarus people** 理解民族或文化差异

*Tao adapted some of the SAS statements to match Chinese culture, for example, the items 'worship' and 'political system' were dropped as potentially misleading (Tao, 2012). The Cronbach's Alpha coefficient for the scale was 0.905*
