# Supplementary material for: Smartphone addiction and cross-cultural adjustment among overseas Chinese students: The role of emotion regulation beliefs and strategies
Source: Front Psychol. 2022 Oct 10;13:1009347. doi: 10.3389/fpsyg.2022.1009347 (PMC9590311; doi:10.3389/fpsyg.2022.1009347)
Supplement: Supplementary file 2 [file Table_2.docx]

(Attachment 2 ) Mobile Phone Addiction Tendency Scale - Chinese, Russian and English versions

| **Chinese simplified** | **Russian (preliminary)** | **English (final version)** |
| --- | --- | --- |
| 指导语：下面是关于手机使用感受的描述,请根据与您实际情况的符合程度,选择一个选项。   - 很不符合 - 不太符合 - 不确定 - 比较符合 - 完全符合 | **Инструкция:** Ниже приводится описание опыта использования мобильного телефона. Пожалуйста, выберите вариант в зависимости от степени соответствия вашей реальной ситуации.   - Совершенно не подходит - Не подходит - Не уверен - В некоторой степени подходит - Полностью подходит | Guidance: The following is a description of the experience of using the mobile phone. Please select an option according to the degree of conformity with your actual situation.   - Very Disagree - Not Appropriate - Not Sure Somewhat - Appropriate - Exactly Appropriate |
| 1一段时间没有带手机我会马上去查阅是否有短信/未接来电。  2我宁愿选择手机聊天，不愿直接面对面交流。  3在等人时我总是频繁打手机问对方身在何处，如果不打就焦急难耐。  4如果很长时间没用手机，我会觉得难受。  5课堂上，我会因为电话或短信而不能专心听讲。  6如果没有手机我会感到孤独。  7我用手机与他人进行交流时，感到更自信。  8一段时间手机铃声不响，我会感到不适应，并下意识看一下手机是否有未接电话/短信。  9我经常有“我的手机铃声响了/我的手机在震动”的幻觉。  10电话多短信多我会觉得生活更充实。  11我经常害怕手机自动关机。  12手机是我的一部分，一旦减少，就觉得失去了什么似的。  13同学朋友常说我太过依赖手机。  14手机经常连不上线、收不到信号时，我会焦虑并且脾气变得暴躁起来。  15课堂上，我会经常主动把注意力集中在手机上而影响听课。  16我觉得用手机跟他人交流更舒适 | 1 Если я какое-то время не брал с собой сотовый телефон, я немедленно проверю, есть ли текстовые сообщения/пропущенные звонки.  2 Я предпочитаю разговаривать по мобильному телефону, чем личное общение.  3 Когда кого-то жду, я всегда часто звоню по телефону, чтобы спросить, где они.  4 Если я долго не пользуюсь телефоном, я чувствую себя некомфортно.  5 В классе я не смогу сосредоточиться на учебе из-за телефонных звонков или текстовых сообщений.  6 Мне одиноко без мобильного телефона.  7 Я чувствую себя более уверенно, когда использую свой телефон для общения с другими людьми.  8 Когда телефон не звонит какое-то время, я чувствую себя некомфортно и незаметно проверяю телефон на наличие пропущенных вызовов или текстовых сообщений.  9 Мне часто кажется, что мой телефон звонит или вибрирует.  10 Больше телефонных звонков и больше текстовых сообщений сделают мою жизнь более полноценной.  11 Я часто боюсь, что телефон незаметно выключится  12 Телефон — это часть меня, и как только он уменьшается, я чувствую, что что-то потерял.  13 Мои одноклассники и друзья часто говорят, что я слишком много полагаюсь на свой мобильный телефон.  14 Когда мой телефон отключается или в нем пропадает сигнал, я нервничаю и раздражаюсь.  15 В классе я часто стремлюсь к тому, чтобы сосредоточиться на своем мобильном телефоне и не слушать учителя.  16 Мне удобнее пользоваться мобильным телефоном для общения с другими. | 1 If I haven't brought my smartphone for a while, I will immediately check to see if there are any text messages or missed calls.  2 I would rather choose a smartphone chat than face-to-face communication.  3 Waiting for someone, I always call the phone frequently to ask where they are.  4 If I don't use my smartphone for a long time, I feel uncomfortable.  5 In class, I won't be able to focus on my studies due to phone calls or text messages.  6 I feel lonely without my smartphone.  7 I feel more confident when using my smartphone to communicate with others.  8 When smartphone doesn't ring for a while, I feel uncomfortable and discreetly check it for missed calls or text messages.  9 I often feel like my smartphone is ringing or vibrating.  10 More phone calls and more text messages will make my life more fulfilling.  11 I am often afraid of my smartphone automatically shutting down.  12 Smartphone is a part of me, and once it decreases, I feel like I've lost something.  13 My classmates and friends often say that I rely too much on my smartphone.  14 When my smartphone turns off or loses signal, I get nervous and annoyed.  15 In class, I often try to focus on my smartphone and not listen to the teacher.  16 I feel more comfortable using my smartphone to communicate with others |
